# Genealogical Analyses of 3 Cultivated and 1 Wild Specimen of *Vitis vinifera* from Greece

**DOI:** 10.1093/gbe/evad226

**Published:** 2023-12-21

**Authors:** Rachita Srivastava, Christos Bazakos, Maroussa Tsachaki, Danijela Žanko, Kriton Kalantidis, Miltos Tsiantis, Stefan Laurent

**Affiliations:** Department of Comparative Development and Genetics, Max Planck Institute for Plant Breeding Research, Cologne 50829, Germany; Department of Comparative Development and Genetics, Max Planck Institute for Plant Breeding Research, Cologne 50829, Germany; Institute of Plant Breeding and Genetic Resources, ELGO-DIMITRA, Thessaloniki 57001, Greece; Plomari Distillery Isidoros Arvanitis S.A., Plomari 81200, Greece; Department of Comparative Development and Genetics, Max Planck Institute for Plant Breeding Research, Cologne 50829, Germany; Department of Biology, University of Crete, Heraklion 71500, Greece; Institute of Molecular Biology and Biotechnology, FORTH, Heraklion 70013, Greece; Department of Comparative Development and Genetics, Max Planck Institute for Plant Breeding Research, Cologne 50829, Germany; Department of Comparative Development and Genetics, Max Planck Institute for Plant Breeding Research, Cologne 50829, Germany; BioNTech, Mainz, Germany

**Keywords:** grapevine, vinetree of Pausanias, Agiorgitiko, Xinomavro, Mavrotragano, ARG reconstruction

## Abstract

Grapevine (*Vitis vinifera*) has been an important crop with considerable cultural and economic significance for over 2,500 years, and Greece has been an important entry point into Europe for lineages that were domesticated in Western Asia and the Caucasus. However, whole-genome-based investigation of the demographic history of Greek cultivars relative to other European lineages has only started recently. To understand how Greek cultivars relate to Eurasian domesticated and wild populations, we sequenced 3 iconic domesticated strains (‘Xinomavro,’ ‘Agiorgitiko,’ ‘Mavrotragano’) along with 1 wild accession (the vinetree of Pausanias—a historically important wild specimen) and analyzed their genomic diversity together with a large sample of publicly available domesticated and wild strains. We also reconstructed genealogies by leveraging the powerful *tsinfer* methodology which has not previously been used in this system. We show that cultivated strains from Greece differ genetically from other strains in Europe. Interestingly, all the 3 cultivated Greek strains clustered with cultivated and wild accessions from Transcaucasia, South Asia, and the Levant and are amongst the very few cultivated European strains belonging to this cluster. Furthermore, our results indicate that ‘Xinomavro’ shares close genealogical proximity with European elite cultivars such as ‘Chardonnay,’ ‘Riesling,’ and ‘Gamay’ but not ‘Pinot.’ Therefore, the proximity of ‘Xinomavro’ to Gouais/Heunisch Weiss is confirmed and the utility of ancestral recombination graph reconstruction approaches to study genealogical relationships in crops is highlighted.

**Significance**

*Vitis vinifera* (grapevine) was cultivated by humans primarily in the Near East (Transcaucasian region and the Levant) and was introduced into Europe via Greece; however, insufficient knowledge exists about the genetic diversity of Greek varieties of grapevine. In this study, we sequenced 3 cultivated and 1 wild variety of grapevine native to Greece and found that they cluster with cultivars from the ancestral range of the species indicating that Greece maintained lineages originally introduced at the onset of the expansion of *V. vinifer*a throughout the Mediterranean basin. Our results also demonstrate that the ancestral recombination graph–based approach used in this study can potentially resolve complex relations between varieties of unknown origin.

## Introduction

*Vitis vinifera*, popularly known as grapevine, is of fundamental economic and cultural significance in human societies because of producing fruits (grapes) for direct consumption or making wine. Wine and table grapes from different parts of the world are prominently distinguishable with respect to phenotypes of agricultural interest, and the effect of latitudinal and longitudinal variation on those phenotypes has been the focus of much attention (This et al. 2006; Laucou et al. 2018; Riaz et al. 2018). Several studies have explored the demographic history of grapevine and suggest that the domestication of *V. vinifera* took place ∼11,000 years ago, during the Neolithic era in Western Asia and the Caucasus, that yielded table and wine grapevines (Dong et al. 2023), whereas previous studies placed the date of the first domestication of grapevine much earlier to 7,000 to 8,000 years ago (Myles et al. 2011; McGovern et al. 2017). Postdomestication, the grapevine spread in 3 directions: Central and East Asia, North Africa, and Europe (Griffith 2004; Myles et al. 2011; McGovern et al. 2017; Zhou et al. 2017; Grassi and Arroyo-Garcia 2020). Grapevine was established in Europe within the last 3,000 years (Xiao et al. 2023), and historic evidence shows extensive population movements across the Mediterranean area, the Caspian Sea area, and the Near East as far as the borders of the Achaemenid (Persian) Empire and the Empire of Alexander the Great. Grapevine movements followed similar paths, indicating Greece was an important hub for introduction and exchange of *V. vinifera* plant material between the European and Asian continents (Negrul 1946; Lefort and Roubelakis-Angelakis 2001).

Grapevine is one of the major crops in Greece and counts more than 600 cultivars in its ampelographic collections, of which 139 are cultivated for table grapes, raisins, and wine (Kotinis 1985; Stavrakas 2010). Despite the long history of Greece in the development of viticulture and its contribution to the dispersal of *V. vinifera* cultivars throughout the expanding Greek territories in the Mediterranean basin since 800 Bc (Lefort and Roubelakis-Angelakis 2001; McGovern and Michel 2003), modern population genetic analysis of major Greek cultivars is scarce leaving many open questions on *Vitis* domestication (but see Forni (2012); Magris et al. (2021); Dong et al. (2023)). In this work, we resequenced the genomes of 3 Greek red grape cultivars and a historical wild vine specimen. Firstly, we sequenced the indigenous Greek cultivar ‘Agiorgitiko,’ which is mainly cultivated in Nemea (Northeastern Peloponnese), one of the largest Greek wine zones and known for its high-quality “protected designation of origin” (PDO) red wines (Lambert-Gócs 1990; Manessis and Anagnostakis 2001; Petropoulos et al. 2018). Secondly, we sequenced the late-ripening indigenous cultivar ‘Xinomavro’ of Northern Greece, which produces PDO wines with long ageing potential due to the phenolic richness and high acidity of the grapes (Kyraleou et al. 2015; Wolkovich et al. 2018). Thirdly, the indigenous cv. ‘Mavrotragano’ is cultivated in a small scale, mainly in the volcanic Cycladic islands of Santorini and Therasia. While the soil of the volcanic islands is overall infertile owing to low clay composition, it also protects the vine plants against phylloxera, a worldwide pest that required most commercial varieties to be grafted on resistant rootstock. As a consequence, own-rooted ‘Mavrotragano’ flourishes and produces exceptional wines with distinctive sensory properties and chemical composition (Stavrakas 2010; Karimali et al. 2020). Finally, the “vine of Pausanias” is a sizeable ancient wild vine that is located by the village of Pagrati in the Central Peloponnese in the courtyard of a small church (37°49′36.96″N, 22°09′16.53″E, alt.: 473 m). The name of this vine plant is attributed to the Greek geographer Pausanias (115 to 180 Ad), who described the presence of a huge vine specimen at this location during his trip in the area (Logothetis 1974; Banilas et al. 2009; Boursiquot et al. 2013). Although no historical data exist to estimate the age of the vine specimen that exists in the same location today, the spectacular size of this plant suggests that it is several hundreds of years old (Boursiquot et al. 2013). An ampelographic analysis of the specimen that we present here concluded that the existing vine of Pausanias is a wild male individual (Boursiquot et al. 2013). The male sex was also confirmed in the present study by genotyping the sex determination region (Massonnet et al. 2020) of the existing vine of Pausanias.

To determine the relationships of Greek varieties within the European cultivated germplasm and to shed light on the origin of the historically relevant “vine of Pausanias,” we conducted joint analyses of 4 new fully sequenced Greek strains together with publicly available strains of both cultivated and wild populations (Zhou et al. 2017, 2019; Liang et al. 2019). We show that Greek strains play a central role in the genealogy of cultivated vines. Moreover, Greek strains provide new evidence for the Balkanic origin of various cultivated varieties of Europe and highlight genetic exchange between South Asia and Europe via Greece.

## Results

**Fig. 1.**
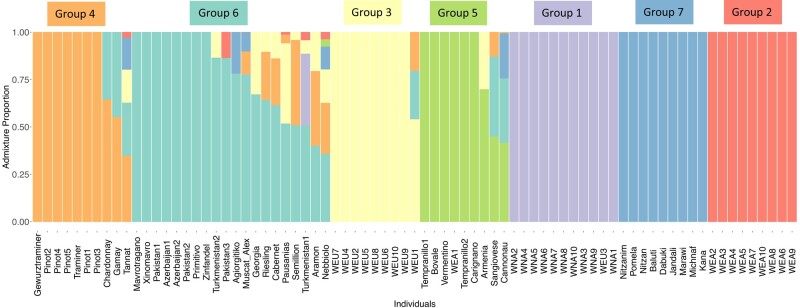
Seven genetic clusters were identified using the software admixture. The optimum number of genetic clusters was chosen by Evanno's method. The first 3 clusters correspond to wild accessions from North America (Group 1), East Asia (Group 2), and Europe (Group 3). Cultivated accessions from France and Germany constitute the 4th cluster (Group 4). The 5th cluster is composed of accessions typical of the Mediterranean range (Group 5) and 1 wild accession from Armenia. Wild accessions from the Transcaucasian region (Azerbaijan, Georgia) and Pakistan and wild and cultivated accessions from Israel constitute the 6th genetic cluster (Group 6). The 7th cluster comprises of wild and cultivated accessions from the Levant region (Israel) (Group 7).

In total, 59,097,679 single-nucleotide polymorphisms (SNPs) were identified by mapping short reads from 77 accessions (supplementary table S1, Supplementary Material online) of wild and cultivated grapevine (Materials and Methods). Admixture analyses of Greek strains along with a subset of publicly available strains from Liang et al. (Liang et al. 2019) and Zhou et al. (Zhou et al. 2017, 2019) (see Materials and Methods) identified 7 genetic clusters (Fig. 1, supplementary fig. S3, Supplementary Material online). The first 3 groups were exclusively composed of wild *V. vinifera* wild strains from North America, East Asia, and Europe (only approximate geographic information was available for strains from Liang et al. (2019)). Group 4 was exclusively composed of cultivated strains typical of France and Germany (‘Gewürztraminer’, ‘Traminer’, ‘Pinot’, ‘Chardonnay’, ‘Gamay’, ‘Tannat’) that correspond to Negrul's *occidentalis* (Negrul 1946). Group 5 was composed of 1 wild accession from Transcaucasia (Armenia) and domesticated varieties typical of the Mediterranean area (‘Tempranillo’, ‘Carignano’, ‘Cannonau’, ‘Vermentino’, ‘Bovale’, ‘Sangiovese’). Group 6 was composed of wild strains from Transcaucasia and Pakistan, as well as domesticated strains from France (‘Riesling’, ‘Cabernet’, ‘Semilion’), Africa (‘Muscat of Alexandria’), and Italy (‘Nebbiolo’, ‘Primitivo’ and ‘Zinfandel’). Unlike the other 6 groups, all but 8 strains from group 6 displayed some level of admixture ranging from 35% to 90%—mainly from the wild European (group 3), group 4, and group 7. Group 7 was composed exclusively of wild and domesticated strains from the Levant region (Israel). These results recapitulate well-known relationships between wild and cultivated lineages (De Michele et al. 2019; Freitas et al. 2021), such as the close proximity of most cultivated lineages with wild Transcaucasian and Levantine populations (Lichter 2005 ; Zohary and Hopf 2000; Riaz et al. 2018). The 3 cultivated Greek strains sequenced in this study (‘Agiorgitiko’, ‘Xinomavro’, and ‘Mavrotragano’) clustered within group 6 and were characterized by a unique ancestry profile among cultivated strains with ∼100% of ancestry of group 6 for ‘Mavrotragano’ and ‘Xinomavro’ and ∼75% and 25% from groups 6 and 7 (Levantine), respectively, for ‘Agiorgitiko’.

**Fig. 2.**
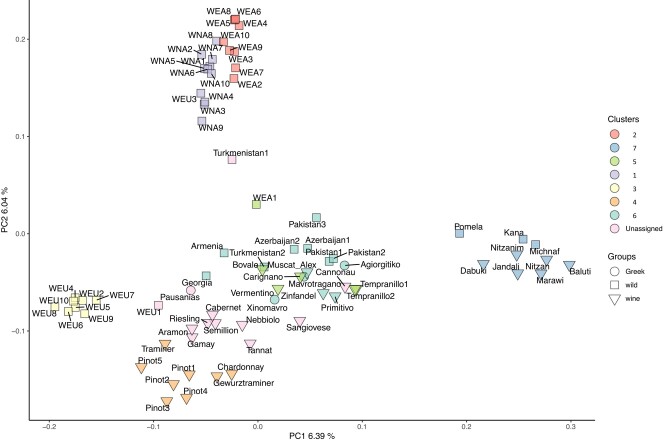
PCA. The first principal component depicts difference between wild (square) and cultivated (triangular) accessions. The second principal component mainly separates cultivated northern cultivated accessions of Europe from the rest. Greek (circular) cultivated accessions along with ‘Primitivo’, ‘Zinfandel’, and ‘Tempranillo’ cluster with wild and cultivated accessions from Levantine region. Accessions in the PCA plot are color coded according to the ancestry groups identified in the admixture analysis (Fig. 1). The accessions with proportions less than 0.65 for all ancestry groups were categorized as unassigned.

The principal component analysis (PCA) of the same set of strains was consistent with the results obtained with *admixture* (Fig. 2). The first principal component mainly reflected differences within cultivated varieties and also isolated the wild European population (WEU). The second principal component mainly separated wild North American and wild East Asian accessions from wild and domesticated *V. vinifera* accessions and further separated cultivated northern European strains (‘Pinot’, ‘Gewürztraminer’, ‘Chardonnay’) from wild populations and also highlighted variation within northern strains. European cultivated strains from group 4 did not display proximity with wild Europeans from group 3 (Ketsch island, Germany). This observation together with the large *F*_ST_ values between group 4 and the other 5 groups (supplementary table S2, Supplementary Material online) is consistent with the idea that the varieties from group 4 (‘Pinot’, ‘Traminer’, ‘Gewürztraminer’, ‘Chardonnay’, and ‘Tannat’) were domesticated from wild lineages that are not present in the set of available wild populations or, alternatively, with high levels of positive selection to the European cultivated group. Also, the intermediate position of ‘Gamay’ and ‘Riesling’ between group 4 and groups 5 and 6 is consistent with their origin as crosses between ‘Pinot’ (group 4) and ‘Gouais’ (syn. Heunisch, not in our sample), which is probably of Mediterranean origin (Bowers et al. 1999). Similarly, the intermediate position of Cabernet–Sauvignon between group 4 and groups 5 and 6 (Mediterranean and Asian) reflects its hybrid origin between ‘Cabernet franc’ (probably of Mediterranean origin) and ‘Sauvignon’ (probably of North European origin) (Bowers and Meredith 1997). Interestingly, ‘Semilion’, ‘Tannat’, and ‘Aramon’ also have an intermediate position between group 4 and groups 5 and 6, suggesting a possible similar hybrid origin between cultivated varieties from Northern and Southern Europe. Greek strains clustered with other strains of group 6 (Fig. 2). Together with ‘Primitivo’ and ‘Zinfandel’ (which have been shown to be synonyms for identical cultivars used in different geographic regions), and ‘Tempranillo’, the Greek strains were closest to the Mediterranean cluster.

**Fig. 3.**
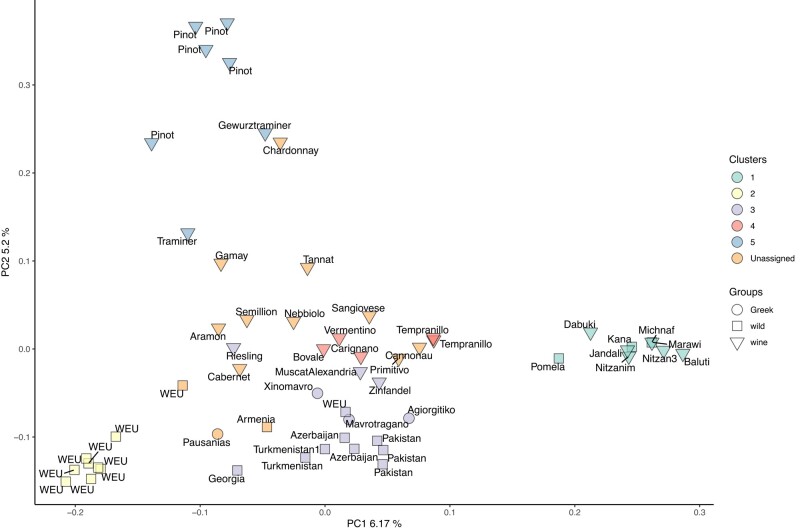
PCA of smaller subset. The first principal component largely highlights the difference between wild European accessions (Cluster 2) and accessions from Israel (Cluster 1). The second principal component as before segregates cultivated accessions from France and Germany (‘Pinot’, ‘Chardonnay’, and ‘Gewurztraminer’) from the rest. Greek accessions (circular) cluster close to wild accessions from the Transcaucasian region. Accessions in the PCA plot are color coded according to the ancestry groups identified in the admixture (supplementary fig. S4, Supplementary Material online). The accessions with proportions less than 0.65 for all ancestry groups were categorized as unassigned.

To evaluate the absolute genetic differentiation between the different ancestry groups, we conducted pairwise *F*_ST_ analyses, a measure to quantify population subdivision based on allele frequencies. Results indicated very low levels of differentiation between group 5, 6, and 7 but larger genetic differentiation between group 4 and the other cultivated groups and to the set of wild European strains analyzed here (*F*_ST_ = 0.34). Because Greek strains did not show any evidence of ancestry related to wild North American and East Asian populations, we decided to reconduct the admixture analysis after excluding them (supplementary fig. S4, Supplementary Material online). The results did not change qualitatively, but the respective proportion of wild European and Transcaucasian ancestry of the “vine of Pausanias” changed slightly. The PCA of this smaller data set indicated that Greek strains clustered very close to the wild Transcaucasian strains (Georgia, Armenia, and Azerbaijan), underscoring the importance of ancient Greece as an entry point for *V. vinifera* into the European continent (Fig. 3).

**Fig. 4.**
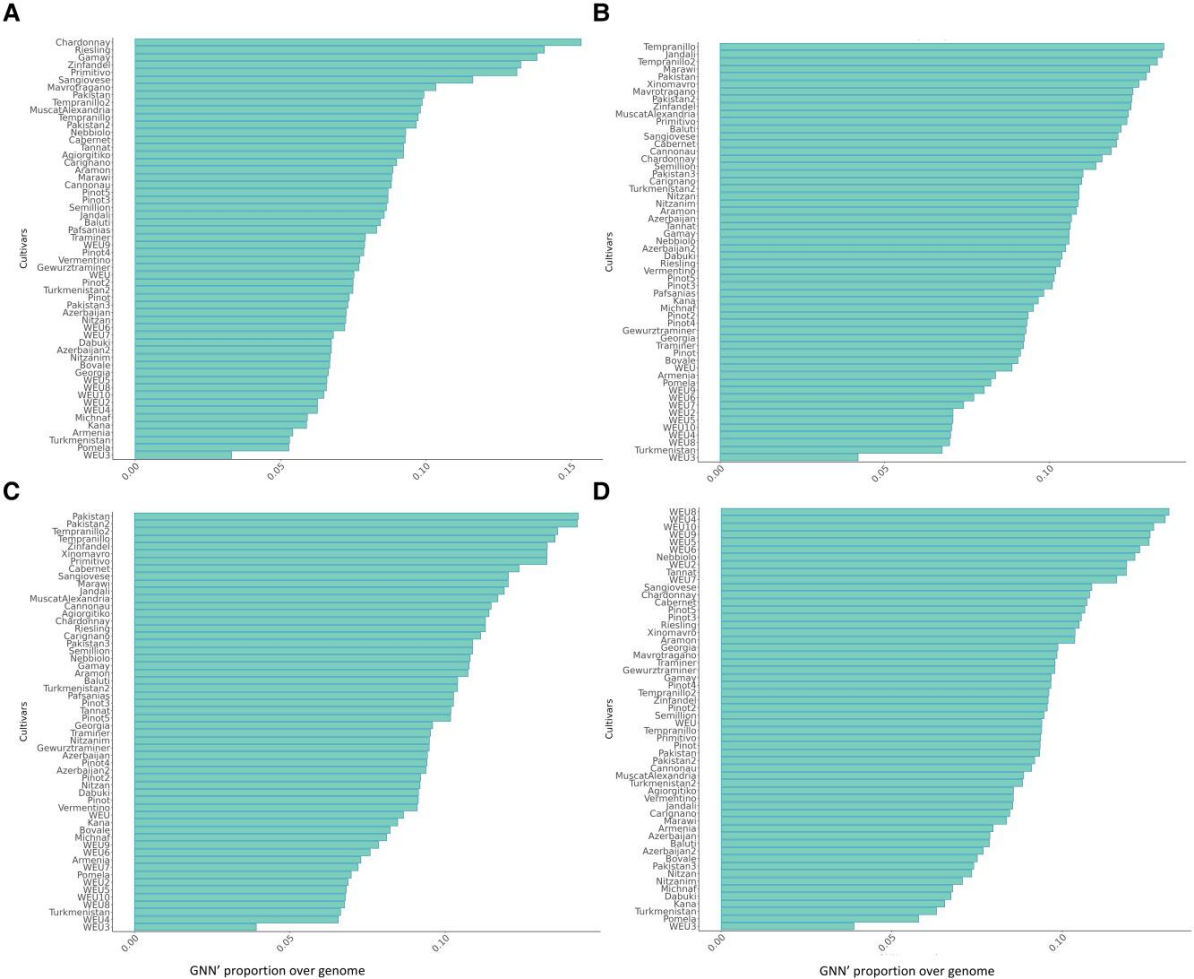
Analyses based on the ARG of the sample presented in Fig. 3. A) GNN′ for ‘Xinomavro’, B) GNN′ for ‘Agiorgitiko’, C) GNN′ for ‘Mavrotragano’, and D) GNN′ for vinetree of Pausanias. The number on the *x* axis (Pu, *x*) represents the proportion of the genome for which the accession was among the closest neighbors to the Greek grapevines of interest (see Materials and Methods).

To obtain a more precise quantification of the ancestry of Greek traditional strains, we reconstructed the genome-wide ancestral recombination graph (ARG) using *tsinfer* (Kelleher et al. 2019). This recently introduced and powerful method allows the reconstruction of the complex genealogical relationships between a sample of fully sequenced chromosomes while accounting for the effect of recombination. *tsinfer* infers the ARG as a sequence of trees along the chromosomes, where the boundaries between successive trees correspond to the genomic locations of inferred recombination breakpoints. We used the ARG obtained with *tsinfer* on each sample shown in Fig. 3 to calculate the distribution of their closest neighbors along all successive trees for each of the 4 Greek strains, an approach we called *GNN*′ (see Materials and Methods). *GNN*′ is similar to the *GNN* method described in Kelleher et al. (2019) with the difference that it calculates how often a single accession (i.e. sequence) is found among the closest neighbors rather than the proportions of different ancestry groups (supplementary fig. S5, Supplementary Material online), as in the case in Kelleher et al. (2019). We also use GNN′ to calculate the proportion of the genome for which 2 accessions are genealogical neighbors. To our knowledge this is the first time that this approach has been used in *V. vinifera*. When applied to ‘Xinomavro’ (Fig. 4, supplementary fig. S6, Supplementary Material online), the *GNN*′ approach revealed that, for ∼15% of its genome, its closest neighbors are ‘Chardonnay’ and ‘Riesling’. Interestingly, previous genetic studies have revealed that ‘Chardonnay’ and ‘Riesling’ derive from crosses involving 1 common parent: the ‘Gouais’ (or ‘Heunisch’), a variety which has almost disappeared today but which was widespread during medieval times and was identified as the progenitor of a large number of European varieties (Bowers et al. 1999). Pedigree analyses based on a small number of SSR markers identified ‘Gouais’ as one of the parents of ‘Xinomavro’; the other parent was unknown (www.vivc.de). Our whole-genome ARG analyses confirm the close relationship between ‘Xinomavro’ and ‘Gouais’ and also suggest a relationship to ‘Zinfandel’/‘Primitivo’, which have also been shown to have a Balkan origin (Calò et al. 2008), and ‘Sangiovese’, which has been recently associated with the germplasm of *Magna Graecia* (a historical denomination for Southern Italian coastal areas under ancient Greek occupation around 800 BC) (De Lorenzis et al. 2020). To gain more insight into the ancestry of ‘Xinomavro,’ we searched for its closest neighbors for each of the 19 chromosomes separately. Our results show that 13 out of the 19 chromosomes had a closest neighbor known to derive from Gouais/Heunisch (‘Chardonnay’, ‘Gamay’, ‘Riesling’; supplementary fig. S6A, Supplementary Material online). For ‘Agiorgitiko’, we identified the Spanish variety Tempranillo as the closest neighbor for ∼12% of the complete genome. However, among closest relatives in the sample, we also identified Jandali, a cultivated variety indigenous to the Southern Levant, supporting the idea that this variety traces back its ancestry both to the Transcaucasian and Levantine areas (supplementary fig. S6B, Supplementary Material online). For ‘Mavrotragano’, the most frequent closest neighbors were 2 wild strains collected in Pakistan and ‘Tempranillo’ (true for 10 out of 19 chromosomes). This is interesting because ‘Mavrotragano’ is thought to have originated on the Greek island of Santorini and does not, to our knowledge, derive from a cross involving the Spanish elite cultivar. It is, therefore, possible that ‘Mavrotragano’ actually originated from an ancient introduction of wild Asian material or vice versa (supplementary fig. S6C, Supplementary Material online).

We found that the most frequent closest genealogical neighbor of the “vine of Pausanias” in our sample was dominated by wild European accessions (WEU) (closest for 9 out of 19 chromosomes; supplementary fig. S6D, Supplementary Material online). To confirm the previous ampelographic analysis (Boursiquot et al. 2013) that the existing “vinetree of Pausanias” accession is indeed a wild male specimen, we conducted genetic analyses of the sex-determining region (SDR) (Massonnet et al. 2020). Several previous studies have identified and extensively studied the SDR by comparing whole-genome sequencing data between the wild (*V. vinifera* ssp. *sylvestris*) and cultivated (*V. vinifera* ssp. *sativa*) grapevine (Massonnet et al. 2020). In *V. vinifera*, SDR spans about 150 kb on chromosome 2 and has been shown to be responsible for the difference in sex type between wild and domesticated varieties of vine (Massonnet et al. 2020). Recent evidence suggests that the loss of dioecy occurred during the domestication process through a rare recombination event between male and female haplotypes that allowed grape growers to enhance the consistency of annual fruit production and remove the necessity for neighboring pollinator vines (Badouin et al. 2020; Massonnet et al. 2020; Zou et al. 2021). Therefore, the sex of a specimen can be determined based on observed polymorphisms at the sex-determining locus (supplementary fig. S7, Supplementary Material online) and we sought to confirm the sex of this accession using our new genomic data for the “vinetree of Pausanias.” Table 1 describes the number of sites with genotypes for male and female alleles and shows the number and state (heterozygous or homozygous) of male and female genotypes at male and female loci as reported earlier (Massonnet et al. 2020). The presence of a high number of dominant alleles at the male locus is indicative of an active male locus (male fertility), and the presence of a high number of dominant (female sterility) compared with the very small number of homozygous recessive alleles (female fertility) at the female locus is indicative of an inactive female locus.

**Table 1:** The number of variants that were identified in the genomic sequence of vinetree of Pausanias against the SDR of the *Vitis vinifera* ssp *vinifera* Cabernet–Sauvignon chromosome 2 hap1 (H) reference (Massonnet et al. 2020) and their classification to dominant or recessive male and female alleles

Mm/MM (active male locus/male fertility)	mm (inactive male locus/male sterility)	Ff/FF (inactive female locus/female sterility)	ff (active female locus/female fertility)
1087	21	225	28

## Discussion

Although the importance of ancient Greek civilization for the introduction of vines into Europe has already been demonstrated (De Lorenzis et al. 2020), evaluation of the relationships between Greek and European strains using fully sequenced Greek genomes is still in its infancy (e.g. (Magris et al. 2021)). Our genetic analyses of ‘Xinomavro’, ‘Agiorgitiko’, and ‘Mavrotragano’ jointly with a large panel of publicly available *sylvestris* and *vinifera* strains indicate that cultivated Greek strains have a central position in the genealogy of cultivated vines. The key role of Greece as an important hub for Vitis introduction to West Europe is also suggested by the mixture of cultivated European varieties and several eastern wild strains (Pakistan, Turkmenistan, Azerbaijan, Georgia) within the same genetic cluster of Greek strains. Such a mixture of wild and domesticated strains was not observed in the other clusters, which were either strictly cultivated or wild (with the exception of a single Wild East Asia (WEA) accession and 1 wild from Armenia in group 5 [Fig. 1]). This is because cluster comembership is indicative of closer genealogical relationships between strains. Interestingly, the clustering of cultivated varieties with Greek strains highlights the 2 geographical routes through which *vinifera* has likely been introduced into the European continent. First, the genealogical proximity of Greek strains with ‘Primitivo’ (and its synonym ‘Zinfandel’) is consistent with the recent discovery of the Balkanic origin of this popular Italian variety (Calò et al. 2008). Similarly, the presence of ‘Riesling’, ‘Chardonnay’, ‘Aramon’, and ‘Gamay’ in the same group as ancient Greek varieties is consistent with the hypothesis of the Balkan origin of the ancient variety ‘Gouais’, which was not directly sequenced in this study but has been shown to be 1 of the 2 parents involved in the crosses from which ‘Riesling’, ‘Chardonnay’, ‘Aramon’, and ‘Gamay’ originated (Regner et al. 1998; Sweet 2009). Thus, these results shed new light on the relationship between Greek and economically important Western varieties. Another interesting relationship was identified based on the proximity of ‘Xinomavro’ and ‘Agiorgitiko’ with ‘Sangiovese’, which appears among the close neighbors of ‘Xinomavro’ and ‘Agiorgitiko’ when GNN′ proportions are averaged over all chromosomes (Fig. 4) and which has recently been shown to have originated in Southern Italy (De Lorenzis et al. 2020). Southern Italy was colonized by Greeks (8th century Bc) (De Lorenzis et al. 2019) who introduced several Greek cultivated varieties on the Italian peninsula. The close genealogical proximity of Sangiovese with Greek strains is therefore consistent with the idea that the ancestor of Sangiovese was introduced by Greek colonizers during the establishment of *Magna Graecia*, as it is already culturally known for varieties such as ‘Aglianico’, ‘Malvasia Nera’, ‘Malvasia di Candia Aromatica’, ‘Malvasia Bianca’, ‘Greco di Tufo’, and ‘Moscato’ (De Lorenziset al. 2020). The presence of ‘Muscat of Alexandria’, cultivated in the Greek island of Lemnos for many hundreds of years, in group 6 with the other cultivated Greek strains is not surprising as the Greek origin of this accession has already been demonstrated before (Lanaridis et al. 2002). Finally, the proximity between ‘Agiorgitiko’ and ‘Jandali’ is an indication that Levantine populations have likely contributed to the original germplasm from which South European cultivated varieties derive.

In addition to ‘Xinomavro’, ‘Agiorgitiko’, and ‘Mavrotragano’, we also report here the genome sequence of the “vinetree of Pausanias” (supplementary fig. S8, Supplementary Material online), a historically important specimen located by the village of Pagrati, about 140 km from Athens. While myths about the origin of this specimen associate it with the Greek historian Pausanias (115 to 180 Ad), a multimillennial age, and early grape production, a scientific study based on ampelographic and genetic analyses of a small number of markers concluded that this specimen is a male *Vitis sylvestris* that does not trace back to any ancient cultivated variety nor could it possibly carry grapes (Boursiquot et al. 2013). However, it is likely that another larger, female individual existed on the site that is now extinct (Logothetis 1974). Although our determination of the sex, by genotyping the SDR (Massonnet et al. 2020), of this accession agrees with the results of Boursiquot et al. (2013), our analyses now also reveal that its ancestry traces back to 2 genetic groups: European sylvestris (WEU, yellow in supplementary fig. S4, Supplementary Material online) and the group containing the cultivated Greek as well as the wild Eastern accession (purple in supplementary fig. S4, Supplementary Material online). Interestingly, our GNN′ analyses indicate that the closest neighbors were ‘Tannat’, ‘Sangiovese’, and ‘Chardonnay’ (Fig. 4, supplementary fig. S6D, Supplementary Material online), suggesting a closer relationship of this specimen to cultivated varieties rather than 2 wild accessions from group 6 (Fig. 1) in our sample. The chromosome-specific GNN′ analysis even revealed that 5 out of 19 chromosomes had their highest *P*_xy_ value for ‘Tannat’ or ‘Riesling’—a pattern that could be explained by genealogical proximity to Gouais/Heunisch or a closely related wild individual.

In conclusion in this work, we clarify the genealogy of 4 iconic Greek vine varieties and confirm recent results identifying a Balkanic ancestry cluster (CG1-containing Greek accessions) as the first specifically European population of *V. vinifera* following divergence from the original Asian population (CG3) (Dong et al. 2023). We also show how genome-wide reconstruction of ARGs can be used as a complementary approach to admixture and PCA analyses in studies of genomic variation of wild and cultivated populations of crop species.

## Materials and Methods

### Plant Material and DNA Sequencing of the 4 Greek Strains

The Vine Nursery Bakasieta (VNB—https://www.bakasietas.gr) provided us with certified vine plants of the 3 Greek cultivars (‘Agiorgitiko’, ‘Xinomavro’, and ‘Mavrotragano’). Additionally, young leaves were collected from the “vine of Pausanias”—a wild vine specimen located in the municipality of Kalavrita, Greece (37°49′37.0″N 22°09′15.6″E). High-quality DNA was extracted with DNeasy PowerPlant Pro Kit (Qiagen Inc., Valencia, CA) from young leaves according to the manufacturer's instructions. The 4 genomic DNA samples were used to generate DNA libraries according to standard Illumina protocols with a mean insertion size of 500 bp. The libraries were sequenced with 150 bp paired-end reads (PE150) using an Illumina HiSeq 3000 system (Illumina, San Diego, CA, United States).

### Read Preprocessing

The raw sequencing data of the 4 Greek genotypes and the fastq files from BioProject PRJNA393611 (Liang et al. 2019), PRJNA388292 (Zhou et al. 2017), and PRJNA550461 (Zhou et al. 2019) were subjected to NGSQCToolkit (Patel and Jain 2012) to remove reads with <25 PHRED quality score. In order to reduce the size of data set for further analysis, we randomly selected 10 strains of each of the wild groups defined in Liang et al. (2019). Supplementary table S1, Supplementary Material online contains the list of Sequence Read Archive (SRA) IDs of publicly available data sets which were analyzed in this study. This filtering produced the high-quality “final raw data.”

Because of a large difference in coverage between our 4 Greek samples and the publicly available data (supplementary fig. S1, Supplementary Material online), we reduced the coverage of the Greek strains to ∼15× by retaining 27,000,000 reads using seqtk (https://github.com/lh3/seqtk) to avoid creating a batch effect in the clustering analyses with the increased SNP calling precision in a subset of the data (Song et al. 2016).

### Read Mapping and Variant Calling

The filtered fastq files were mapped to the ‘Pinot’ Noir reference sequence PN40024 (Jaillon et al. 2007) with *bwa mem v0.7.17* (Li 2013), and the resultant SAM files were converted to BAM using *samtools* sort (Li et al. 2009). Duplicated reads were removed from BAM files using *picardtools markduplicates* (https://broadinstitute.github.io/picard/). Mean depth of each sample was calculated using *samtools* depth (Danecek et al. 2021) (supplementary fig. S1, Supplementary Material online). Genotype likelihoods for the BAM files were calculated using *ANGSD* (Korneliussen et al. 2014) with *-GL 2 -doGlf 2 -doMajorMinor 1 -SNP_pval 1e-6 -doMaf 1* options, and a relatedness analysis was conducted based on the genotype likelihoods using *ngsrelate* (https://github.com/ANGSD/NgsRelate). Variant calling was done using *gatk v4.1.7* (Poplin et al. 2018) following best practices published in the GATK manual. *Snakemake* (Mölder et al. 2021) was used to implement the workflow for reproducibility, and snakemake files can be made available upon request. The variant file was filtered with *gatk Select Variants* such that SNPs satisfying the following criteria were discarded: QD < 2.0, QUAL < 30.0, SOR > 3.0, FS > 60.0, MQ < 40.0, MQRankSum < −12.5, and ReadPosRankSum < −8.0.

### Admixture Analysis

The variant file was pruned for linkage disequilibrium using *plink v2.00a* (Chang et al. 2015) --indep-pairwise 20 Kb 0.2 after allowing for a maximum of 20% missing data before subjecting it to admixture analysis. *Admixture* (Alexander et al. 2009) was performed for *K* values of 2 to 10, and 75 independent runs were carried out for each *K* value. Optimal number of clusters was inferred by Evanno's method using *clumpak* (Kopelman et al. 2015). The best run of the 75 runs of optimum *K* was selected based on lowest Cross-Validation (CV) error. PCA was executed on the pruned file using *SNPRelate* (Zheng et al. 2012). Pairwise *F*_ST_ values for every pair of ancestry groups identified by *admixture* were generated using *VCFtools* (Danecek et al. 2011) with the option *--weir-fst-pop*.

### Neighbor Joining Tree

Distance matrix was constructed based on genome-wide SNPs for all 77 individuals using VCF2Dis (https://github.com/BGI-shenzhen/VCF2Dis). The output distance matrix was utilized as an input for FastME 2.0 (Lefort et al. 2015), and the corresponding output tree was visualized using iTOL (Letunic and Bork 2021) (supplementary fig. S2, Supplementary Material online).

### ARG Analysis (GNN′)

*tsinfer* (Kelleher et al. 2019) was used to construct the ARG from the variant file. Variants were polarized using North American strains as outgroups (WNA, group 1 in Fig. 1) as was done in Zhou et al. (2019). The ancestral allele was defined as the major allele in the WNA sample (*n* = 10), and sites where the major allele was <9 out of 10 were excluded. The variant file for sites with only 2 alleles was phased and imputed based on genetic map (Tello et al. 2019) using *Shapeit v4.0* (Delaneau et al. 2019) before inferring the ARG. The vcf file was split based on 19 chromosomes, and the tree sequence representation of the ARG was generated for each chromosome's variant file for the same set of individuals. GNN′ was then carried out by using 1 of the 4 Greek cultivars as the focal node each time.

### GNN′

Kelleher et al. (2019) described the *genealogical nearest neighbors* (*GNN*) approach, which calculates for any sampled individual the proportions of different ancestry groups among its nearest neighbors. Here, we propose *GNN*′, a modification of this approach that allows calculating the proportion of the genome for which 2 accessions are neighbors.

Let *X* be a set of all leaves of a bifurcating tree (i.e. set of our present-day sample) and let *T* be a set of all the bifurcating trees belonging to a tree sequence. For every focal node u∈X and every bifurcating tree t∈T, we define an indicator function:


$$I_{N_{u,t}}:X\setminus \{u\}\to \{0,1\}.$$

$$\;\;I_{N_{u,t}}(x):\;=\{\begin{array}{cc} 1 & \mathrm{if}\;u\in N\\ 0 & \mathrm{if}\;u\notin N \end{array},$$

where N⊆X∖{u} containing all of the leaves descending from the same *parental node v* as focal node *u*. Formally, *parental node v* of a focal node *u* is the first node on the path from *u* to the root in *t* (see examples in supplementary fig. S5, Supplementary Material online).

For each x∈X∖{u}, we define:


$$\;G_{u,x}:\;=\;\frac{\;\sum_{t\in T}\;I_{u,t}(x)}{T}\;,\;0\leq G_{u,x}\leq 1,$$

where $\sum_{t\in T}I_{u,t}(x)$ represents the total number of trees in a tree sequence for which *u* and *x* are neighbors (i.e. x∈N for a focal node *u*). Dividing by total number of trees in the tree sequence gives a proportion of trees for which *u* and *x* are neighbors. Each tree t∈T covers a span of $L^{t}$ units of genetic sequence (i.e. base pairs), and $L=\sum_{t\in T}L^{t}$, giving us the final measure:


$$\;\;P_{u,x}:\;=\;\frac{1}{L}\underset{t\in T}{\sum }\;L^{t}I_{u,t}(x)\;,\;0\leq P_{u,x}\leq 1.$$

$P_{u,x}$ measures the proportion of the genome for which *u* and *x* are neighbors.

### Sex Determination Analysis

For the sex determination of the “vine of Pausanias,” whole-genome sequence reads were mapped to Cabernet–Sauvignon hap1 H haplotype (https://zenodo.org/record/3827985#.YVR_HkZBxN0) (Massonnet et al. 2020). Variants were called using *BCFtools call* (Li 2011). The alleles at the markers reported by Zhou et al. (2019) were examined for their state for homozygosity for reference allele and heterozygosity or homozygosity for the alternate allele.

## Supplementary Material

evad226_Supplementary_Data

## Data Availability

The data underlying this article are available in Genbank at www.ncbi.nlm.nih.gov/genbank and can be accessed with PRJNA856851. All scripts are available on GitLab (https://gitlab.mpcdf.mpg.de/g-rachitasrivastava/vitis_manuscript).
