# Supplementary material for: Genealogical Analyses of 3 Cultivated and 1 Wild Specimen of Vitis vinifera from Greece
Source: Genome Biol Evol. 2023 Dec 21;15(12):evad226. doi: 10.1093/gbe/evad226 (PMC10735296; doi:10.1093/gbe/evad226)
Supplement: evad226_Supplementary_Data [file evad226_supplementary_data.docx]

**Supplementary Figures and Tables**

**Supplementary Figure 1: Mean depth of all samples**

Mean depth of all samples calculated using samtools depth on bam files. The red points for Greek

samples show the original mean depth of the samples and the green points for the same Greek samples

show the mean depth of samples after down-sampling. The blue points represent mean depth for all the remaining 73 samples apart from Greeks. The down-sampling of the Greek lines was done to homogenize the coverage in dataset and avoid potential batch effects in the variant calling.

**Supplementary Figure 2. Neighbour-joining tree based on the distance matrix of SNPs.**

Neighbor joining tree based on distance matrix constructed using VCF2Dis (https://github.com/BGI-shenzhen/VCF2Dis) on genome wide SNPs for 77 accessions. Neighbor joining tree was constructed using Fastme (Lefort, et al. 2015) and the tree was visualized using ITOL (Letunic and Bork 2021). The Greek cultivated and wild accessions are marked in magenta.

**Supplementary Figure 3:** Admixture – choice of K


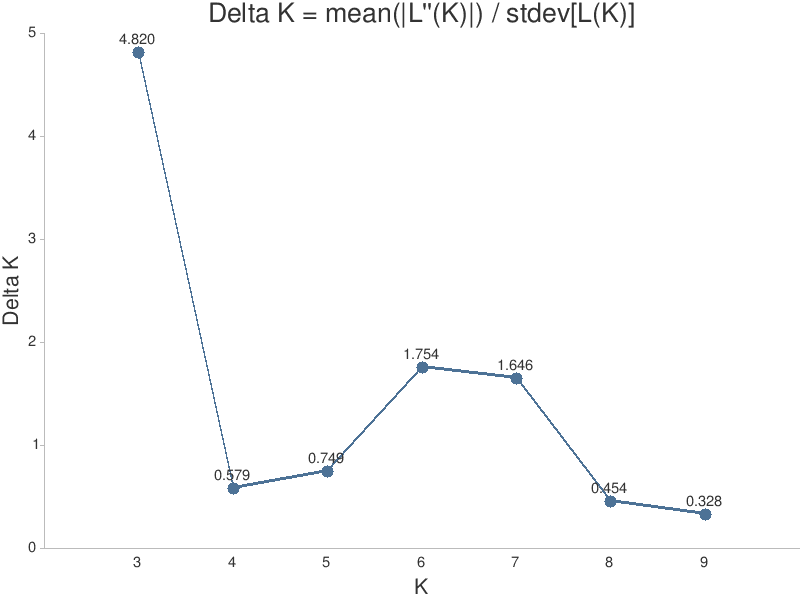


Delta K analysis for choosing the best number of genetic clusters in the admixture analysis. The optimal K was obtained using the method of Evanno et al (Evanno, et al. 2005) which is based on the largest decrease in delta K between two successive values, where K is the number of genetic groups.

**Supplementary Figure 4.**

Admixture analysis on a smaller dataset. This analysis identifies five genetic clusters according to Evanno’s method. The second cluster identifies wild accessions from Europe (yellow). Levantine accessions cluster together (bottle green). Cultivated and wild Greek accessions cluster in group four (purple) with varying levels of admixture with other groups.

**Supplementary Fig 5.**

Examples of two bifurcating trees belonging to a tree sequence depicting the counting schema for genealogical nearest neighbours used in GNN'. A) For focal node u, genealogical nearest neighbours are a,b and c and each of them is assigned a score of 1 for this tree whereas d,e and f are assigned a score of 0 as they are not belong to the list of child nodes of parental node v. B) A) For focal node u, genealogical nearest neighbours is a is assigned a score of 1 for this tree whereas b,c,d,e and f are assigned a score of 0 as they are not belong to the list of child nodes of parental node v. This is different from the counting scheme described in the original algorithm (Kelleher et al (Kelleher, et al. 2019)) and is adapted for the case when the genealogical unit of interest is a single accession (e.g. a cultivar) rather than an ancestry group (as it is the case in Kelleher et al (Kelleher, et al. 2019)).

**Supplementary Fig 6.**

GNN’ (Gealogical nearest neighbour modified) analysis on the ancestral recombination graph for greek samples. The x-axis represents all the chromosomes and y-axis represents the gnn’ proportion of the cultivar which most frequently occurs as the nearest neighbour to the focal cultivar.

**Supplementary Fig 7.**

Schematic representation of genetic basis of sex-determination in *V. vinifera.* Blue block represents an active male locus and pink block represents and active female locus. Inactive male and female locus is symbolized by red block. The state of female and female allele as homozygous or heterozygous determines whether the male or female locus is active/inactive. Image adapted from Massonnet, M., Cochetel, N., Minio, A. et al. The genetic basis of sex determination in grapes. Nat Commun 11, 2902 (2020).

**Supplementary Fig 8.**

Overview of the historical vine tree of Pausanias in April 2019. (A) Trunk and (Korneliussen, et al.) Leaf morphology of the vine tree of Pausanias (*Vitis vinifera* subsp *sylvestris*).

**Supplementary Table 1** Information on the SRA ids of publicly available datasets utilized in this study.

| Accessions | Country | Group | SRA |
| --- | --- | --- | --- |
| Aramon | France | w | SRR5627800 |
| Armenia |  | wild | SRR5627784 |
| Azerbaijan1 |  | wild | SRR5627787 |
| Azerbaijan2 |  | wild | SRR5627790 |
| Baluti | Israel | w | SRR3509718 |
| Bovale Sardo | Sardinia | w | SRR5803837 |
| Cabernet_Sauvignon08 | CA | w | SRR5627795 |
| Cannonau | Sardinia | w | SRR5803836 |
| Carignano | Sardinia | w | SRR5803839 |
| Chardonnay | CA | w | SRR5627799 |
| Dabuki | Israel | w | SRR3528127 |
| Gamay_Noir |  | w | SRR5627798 |
| Georgia |  | wild | SRR5627789 |
| Gewurztraminer | Italy | w | ERR514999 |
| Jandali | Israel | w | SRR3528198 |
| Kana_wild_female | Israel | wild | SRR3509067 |
| Marawi | Israel | w | SRR3509720 |
| Michnaf_wild_female | Israel | wild | SRR3509717 |
| Muscat of Alexandria | North Africa | w | SRR5627781 |
| Nebbiolo_CVT423 | Italy | w | SRR5626750 |
| Nitzan3 | Israel | w | SRR3508973 |
| Nitzanim_Bustan | Israel | w | SRR3496931 |
| Pakistan1 |  | wild | SRR5627785 |
| Pakistan2 |  | wild | SRR5627792 |
| Pakistan3 |  | wild | SRR5627791 |
| Pinot Blanc | France | w | SRR3509362 |
| Pinot Gris | France | w | SRR3509358 |
| Pinot Meunier | France | w | SRR3509365 |
| Pinot Noir |  | w | SRR3990782 |
| Pomela_wild_female | Israel | wild | SRR3497168 |
| Primitivo03 | Italy | w | SRR5627796 |
| Riesling | INRA | w | SRR5627794 |
| Sangiovese | Italy | w | SRR5506711 |
| Semilion | France | w | SRR5627793 |
| Tannat | Uruguay | w | SRR863618 |
| Tempranillo Blanco | Spain | w | SRR2895165 |
| Tempranillo Tinto | Spain | w | SRR2895164 |
| Traminer | Italy | w | SRR5627802 |
| Turkmenistan1 |  | wild | SRR5627783 |
| Turkmenistan2 |  | wild | SRR5627786 |
| Vermentino | Sardinia | w | SRR5803838 |
| Zinfandel_03 | CA | w | SRR5627801 |
| Pinot Noir |  | w | SRR5627797 |
| WNA |  | wild | SRR5891645 |
| WNA |  | wild | SRR5891646 |
| WNA |  | wild | SRR5891649 |
| WNA |  | wild | SRR5891651 |
| WNA |  | wild | SRR5891652 |
| WNA |  | wild | SRR5891653 |
| WNA |  | wild | SRR5891654 |
| WEU |  | wild | SRR5891677 |
| WEA |  | wild | SRR5891684 |
| WNA |  | wild | SRR5891802 |
| WNA |  | wild | SRR5891806 |
| WNA |  | wild | SRR5891919 |
| WEA |  | wild | SRR5891685 |
| WEA |  | wild | SRR5891698 |
| WEA |  | wild | SRR5891758 |
| WEA |  | wild | SRR5891929 |
| WEA |  | wild | SRR5891932 |
| WEA |  | wild | SRR5892013 |
| WEA |  | wild | SRR5892014 |
| WEA |  | wild | SRR5892015 |
| WEA |  | wild | SRR5892016 |
| WEU |  | wild | SRR5891676 |
| WEU |  | wild | SRR5891678 |
| WEU |  | wild | SRR5891679 |
| WEU |  | wild | SRR5891703 |
| WEU |  | wild | SRR5891708 |
| WEU |  | wild | SRR5891709 |
| WEU |  | wild | SRR5891759 |
| WEU |  | wild | SRR5891883 |
| WEU |  | wild | SRR5891607 |

**Supplementary Table 2** Genetic differentiation between all sets of genetic ancestry clusters estimated by admixture

| Pop1 | Pop2 | Weighted Fst |
| --- | --- | --- |
| Cluster1(WNA) | Cluster2(WEA) | 0.19944 |
| Cluster1(WNA) | Cluster3(WEU) | 0.38831 |
| Cluster1(WNA) | Cluster4(North European) | 0.36153 |
| Cluster1(WNA) | Cluster5(Mediterranean) | 0.31788 |
| Cluster1(WNA) | Cluster6(Temperate zone Eurasia) | 0.31052 |
| Cluster1(WNA) | Cluster7(Levant) | 0.32083 |
| Cluster2(WEA) | Cluster3(WEU) | 0.3427 |
| Cluster2(WEA) | Cluster4(North European) | 0.31795 |
| Cluster2(WEA) | Cluster5(Mediterranean) | 0.24845 |
| Cluster2(WEA) | Cluster6(Temperate zone Eurasia) | 0.23266 |
| Cluster2(WEA) | Cluster7(Levant) | 0.2518 |
| Cluster3(WEU) | Cluster4(North European) | 0.34141 |
| Cluster3(WEU) | Cluster5(Mediterranean) | 0.29893 |
| Cluster3(WEU) | Cluster6(Temperate zone Eurasia) | 0.21457 |
| Cluster3(WEU) | Cluster7(Levant) | 0.33682 |
| Cluster4(North European) | Cluster5(Mediterranean) | 0.226 |
| Cluster4(North European) | Cluster6(Temperate zone Eurasia) | 0.14637 |
| Cluster4(North European) | Cluster7(Levant) | 0.24695 |
| Cluster5(Mediterranean) | Cluster6(Temperate zone Eurasia) | 0.033327 |
| Cluster5(Mediterranean) | Cluster7(Levant) | 0.082786 |
| Cluster6(Temperate zone Eurasia) | Cluster7(Levant) | 0.056982 |

Evanno G, Regnaut S, Goudet J 2005. Detecting the number of clusters of individuals using the software STRUCTURE: a simulation study. Mol Ecol 14: 2611-2620. doi: 10.1111/j.1365-294X.2005.02553.x

Kelleher J, et al. 2019. Inferring whole-genome histories in large population datasets. Nat Genet 51: 1330-1338. doi: 10.1038/s41588-019-0483-y

Korneliussen TS, Albrechtsen A, Nielsen R 2014. ANGSD: Analysis of Next Generation Sequencing Data. BMC Bioinformatics 15: 356. doi: 10.1186/s12859-014-0356-4

Lefort V, Desper R, Gascuel O 2015. FastME 2.0: A Comprehensive, Accurate, and Fast Distance-Based Phylogeny Inference Program. Molecular Biology and Evolution 32: 2798-2800. doi: 10.1093/molbev/msv150

Letunic I, Bork P 2021. Interactive Tree Of Life (iTOL) v5: an online tool for phylogenetic tree display and annotation. Nucleic Acids Research 49: W293-W296. doi: 10.1093/nar/gkab301
